# CLASP promotes microtubule bundling in metaphase spindle independently of Ase1/PRC1 in fission yeast

**DOI:** 10.1242/bio.045716

**Published:** 2019-10-15

**Authors:** Hirohisa Ebina, Liang Ji, Masamitsu Sato

**Affiliations:** 1 Laboratory of Cytoskeletal Logistics, Department of Life Science and Medical Bioscience, Graduate School of Advanced Science and Engineering Waseda Research Institute for Science and Engineering, Waseda University, TWIns, 2-2 Wakamatsucho, Shinjuku-ku, Tokyo 162-8480, Japan; 2 Department of Biophysics and Biochemistry, Graduate School of Science, University of Tokyo, 7-3-1 Hongo, Bunkyo-ku, Tokyo 113-0033, Japan; 3 Institute for Medical-Oriented Structural Biology Waseda Research Institute for Science and Engineering, Waseda University, TWIns, 2-2 Wakamatsucho, Shinjuku-ku, Tokyo 162-8480, Japan; 4 Institute for Advanced Research of Biosystem Dynamics, Waseda Research Institute for Science and Engineering, Waseda University, TWIns, 2-2 Wakamatsucho, Shinjuku-ku, Tokyo 162-8480, Japan

**Keywords:** CLASP, Microtubule, Microtubule-associated protein, Mitotic spindle

## Abstract

Microtubules in the mitotic spindle are organised by microtubule-associated proteins. In the late stage of mitosis, spindle microtubules are robustly organised through bundling by the antiparallel microtubule bundler Ase1/PRC1. In early mitosis, however, it is not well characterised as to whether spindle microtubules are actively bundled, as Ase1 does not particularly localise to the spindle at that stage. Here we show that the conserved microtubule-associated protein CLASP (fission yeast Peg1/Cls1) facilitates bundling of spindle microtubules in early mitosis. The *peg1* mutant displayed a fragile spindle with unbundled microtubules, which eventually resulted in collapse of the metaphase spindle and abnormal segregation of chromosomes. Peg1 is known to be recruited to the spindle by Ase1 to stabilise antiparallel microtubules in late mitosis. However, we demonstrate that the function of Peg1 in early mitosis does not rely on Ase1. The unbundled spindle phenotype of the *peg1* mutant was not seen in the *ase1* mutant, and Peg1 preferentially localised to the spindle even in early mitosis unlike Ase1. Moreover, artificial overexpression of Ase1 in the *peg1* mutant partially suppressed unbundled microtubules. We thus conclude that Peg1 bundles microtubules in early mitosis, in a distinct manner from its conventional Ase1-dependent functions in other cell cycle stages.

## Abstract (precis)

**Summary:** The conserved microtubule-associated protein CLASP/Peg1/Cls1 bundles microtubules of the metaphase spindle in fission yeast. Unlike its conventional role in anaphase, this CLASP function does not depend upon Ase1/PRC1.

## INTRODUCTION

The mitotic spindle segregates chromosomes into two daughter cells. The spindle consists of microtubules (MTs) and microtubule-associated proteins (MAPs). MT is a dynamic intracellular component that repeats polymerisation and depolymerisation of α/β-tubulin dimers to assemble a filamentous organization. The cytoplasmic array of MTs is reorganised into the spindle at the onset of mitosis. MTs intensively emanate from two spindle poles such as centrosomes or spindle pole bodies (SPBs). Spindle MTs are then augmented by a number of MAPs promoting MT nucleation, controlling the dynamic property of MTs, and bundling MT filaments. All of those processes are required to establish bipolarity and the robust spindle structure, as failures in either process may result in unequal segregation of chromosomes (McDonald and McIntosh, 1993; McDonald et al., 1992).

MT bundling is particularly essential to stabilise and maintain the structural backbone of the spindle. MTs emanating from each spindle pole are connected through bundling mostly at the centre of the spindle, throughout mitosis. Several proteins promoting MT bundling have been identified to date.

The conserved non-motor MAP Ase1/PRC1 preferentially associates with the overlapping zone of antiparallel MTs at the centre of the spindle to stably bundle them (Janson et al., 2007; Jiang et al., 1998; Loïodice et al., 2005; Mollinari et al., 2002; Yamashita et al., 2005). Ase1/PRC1 exerts its function particularly in late stages of mitosis (anaphase), as localisation and the activity of Ase1/PRC1 in early mitosis (prophase to metaphase; pre-anaphase) is negatively regulated through phosphorylation by the cyclin-dependent kinase Cdk1 (Fu et al., 2009; Jiang et al., 1998; Mollinari et al., 2002). This indicates that antiparallel MTs in early mitosis are not predominantly bundled, and that other mechanisms exist to maintain the spindle robustness during pre-anaphase. Motor proteins are also well characterised bundling factors; the plus-end directed kinesin kinesin-5/Cut7/KIF11/Eg5 associates with two bundles of MTs to slide them apart as it moves toward their plus ends (Blangy et al., 1995; Hagan and Yanagida, 1990, 1992; Le Guellec et al., 1991; Sawin et al., 1992). In general, kinesin-5 is essential in separation of spindle poles to assemble the bipolar spindle in prophase. On the other hand, minus-end directed kinesins including kinesin-14/Plk1/Klp2/HSET mainly tether MTs aligned in parallel, as they move towards the minus end, so that a number of MTs can be firmly focused at spindle poles (Cai et al., 2009; Pidoux et al., 1996; Troxell et al., 2001).

Cooperative functions of those kinesin motor proteins are essential to assemble bipolar spindles. In fission yeast, however, the double knockout strain of both kinesin-5 and kinesin-14 motors is viable and still displays antiparallel MT bundles in the pre-anaphase spindle (Olmsted et al., 2014). This means that additional factors bundle MTs during pre-anaphase in combination with motor proteins. Ase1 is known to be required for spindle pole separation in the absence of both Cut7 and Pkl1 (Rincon et al., 2017; Yukawa et al., 2017). As mentioned above, Ase1 in wild-type (WT) cells does not predominantly localise to the spindle in early mitosis, it is possible that an additional factor to bundle MTs other than Ase1 may exist for pre-anaphase spindle.

Cytoplasmic linker-associated protein (CLASP) is a MAP widely conserved in eukaryotes (Akhmanova et al., 2001; Gönczy et al., 2000; Grallert et al., 2006; Inoue et al., 2000; Lemos et al., 2000; Maiato et al., 2003; Pasqualone and Huffaker, 1994). A number of studies have demonstrated the significance of CLASP in spindle assembly in various organisms (Bratman and Chang, 2007; Espiritu et al., 2012; Hannak and Heald, 2006; Inoue et al., 2004; Maiato et al., 2002; Mimori-Kiyosue et al., 2006; Pereira et al., 2006). The fission yeast CLASP orthologue Peg1/Cls1 localises to the spindle in late mitosis in an Ase1-dependent manner to ensure anaphase spindle integrity (Bratman and Chang, 2007; Grallert et al., 2006). Peg1, however, localises to the spindle even from pre-anaphase, when Ase1 does not predominantly localise to the spindle. This suggests that Peg1 localisation in pre-anaphase may not depend upon Ase1. As the *peg1* mutant shows severe defects in spindle assembly even in pre-anaphase (Bratman and Chang, 2007; Grallert et al., 2006), it may be interesting to pursue the function of Peg1 in pre-anaphase.

Here we demonstrate that Peg1 facilitates MT bundling in pre-anaphase. Peg1 functions independently of Ase1 in pre-anaphase unlike in late mitosis, suggesting that Peg1 is regulated in a cell cycle-dependent manner so that Peg1 can change its behaviour and functions depending upon cell cycle stages. This illuminates a novel mechanism of microtubule bundling in pre-anaphase of mitosis.

## RESULTS

### Spindle MTs were often unbundled in the *peg1-104* mutant during pre-anaphase

To investigate possible involvement of Peg1 during pre-anaphase, we created conditional mutants of the *peg1* gene that showed severe phenotype at the restrictive temperature, as the gene has been shown essential for viability (Grallert et al., 2006). Using PCR-based random mutagenesis, we isolated several *peg1* temperature-sensitive mutants (Fig. S1A). We picked up one of the mutants, *peg1-104*, for further analyses, as it showed more severe temperature sensitivity than others (Fig. S1B). The *peg1-104* mutant harbours a single point mutation, L797P. This was identical to one of the *peg1/cls1* mutants (*cls1-28*) reported previously (Bratman and Chang, 2007).

The green fluorescent protein (GFP) gene was knocked-in at the end of coding sequences of the WT *peg1*^+^ and the *peg1-104* mutant genes to express fusion proteins of Peg1 and Peg1-104 with GFP at the endogenous level, respectively. The WT Peg1-GFP localised to the spindle during mitosis as reported previously (Bratman and Chang, 2007), whereas Peg1-104-GFP did not at the restrictive temperature (Fig. S2A). Immunoblotting assays revealed that the amount of Peg1-104-GFP was comparable to that of WT Peg1-GFP (Fig. S2B,C) at any time. The temperature-sensitive growth defect of the *peg1-104* mutant was suppressed when WT Peg1-mCherry was ectopically expressed (Fig. S2D), consistently indicating that the *peg1-104* mutant is recessive. We adopted the *peg1-104* mutant hereafter.

**Fig. 1.**
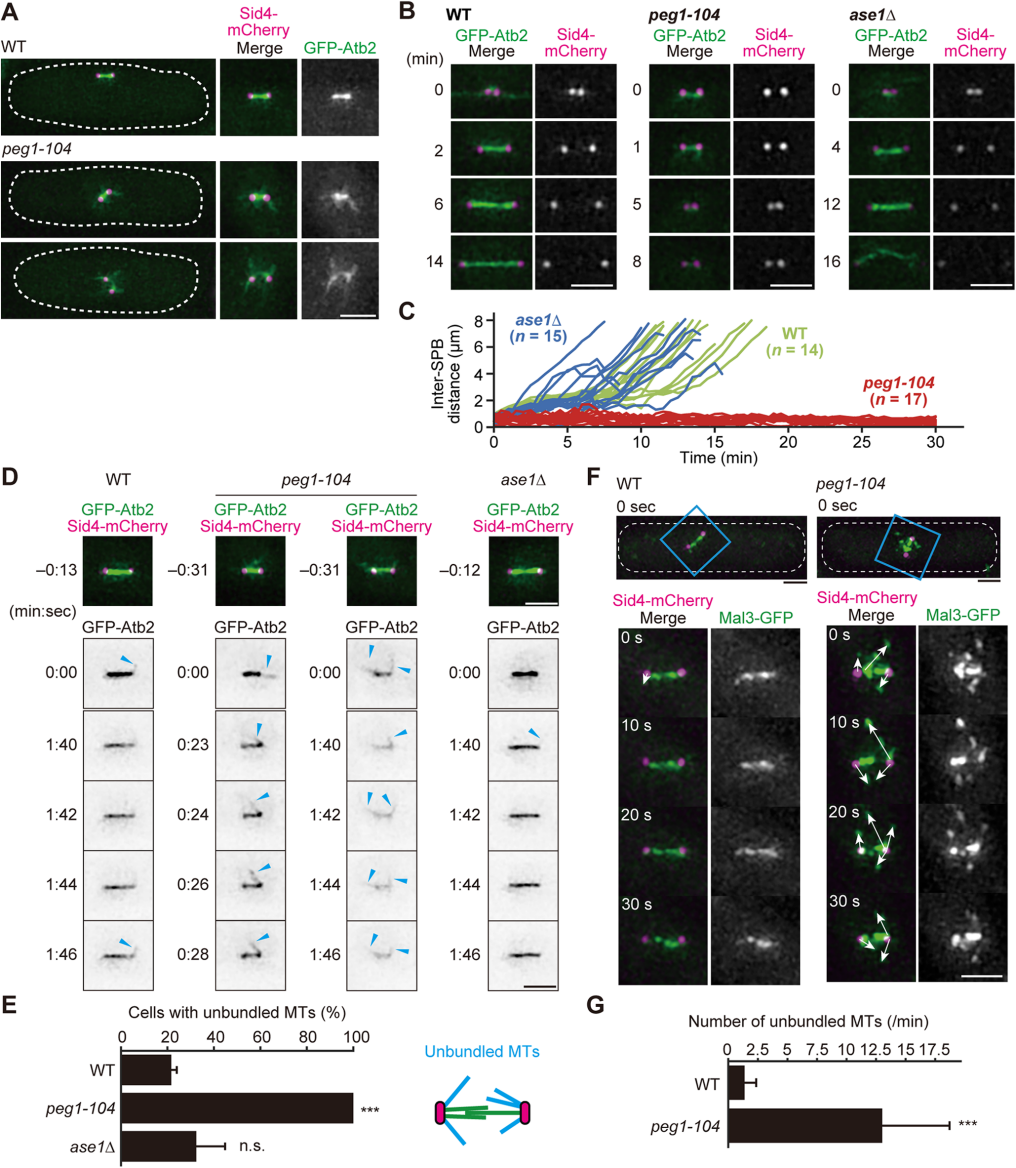
**Spindle MTs are not bundled in the *peg1-104* mutant in pre-anaphase.** (A) Pre-anaphase cells of WT strain and the *peg1-104* mutant expressing GFP-Atb2 (α-tubulin; MT, green) and Sid4-mCherry (SPB, magenta) grown at 32°C for 3 h. The cell shape is outlined. (B,C) Behaviour of the spindle throughout mitosis in living WT, *peg1-104* and *ase1*Δ cells at 32°C (B). Filming started at time 0 (min). Kinetics of spindle length during observation is shown (C). The length is defined as the distance between two SPBs. (D) Visualisation of unbundled MTs in the spindle of indicated strains. Cells were grown at 25°C and shifted to 32°C before observation. Spindles of ∼2 μm were captured with GFP-Atb2 (MT) and Sid4-mCherry (SPB), followed by taking monochromatic images of GFP-Atb2 every 2–3 s. Time (min:s) is shown in each image. Arrowheads indicate unbundled MTs. (E) Frequency of cells displaying the spindle with unbundled MTs during 5-min filming was quantified using the strains in D. Mean of three experiments is shown with error bars, s.d. (*n*=10, 15 and 16 cells, WT; *n*=15, 17 and 11 cells, *peg1-104*; *n*=13, 16 and 18 cells, *ase1*Δ). (F,G) Mal3-GFP (green) and Sid4-mCherry (magenta) were used to visualise MT behaviour in WT and *peg1-104* spindles. Cell shape is outlined, and insets including the pre-anaphase spindle are magnified to show time-lapse images of the area (F). Direction of unbundled MT extension is indicated by arrows. Numbers of unbundled MTs per minute are shown (G). *n*=11 cells. Error bars, s.d.; ***P<0.0005; n.s., no significance; two-tailed Welch's *t*-test. Scale bars: 2 μm.

To further investigate details as to how the spindle is defective in the *peg1-104* mutant, we used the Z2-GFP-Atb2 system to visualise MTs, in which the *GFP-atb2* (α-tubulin) fusion gene is expressed as an extra-copy of the endogenous *atb2*^+^ gene (Ohta et al., 2012). Sid4-mCherry was used as a marker protein for the SPB (the equivalent structure to the vertebrate centrosome). In WT cells, MTs assembled the robust rod-shaped bipolar spindle during pre-anaphase, whereas the spindle was fragile in *peg1-104* cells as previously reported (Bratman and Chang, 2007) (Fig. 1A). We reasoned this was due to defects in MT bundling, as the spindle in the mutant frequently displayed MTs emanating from SPBs towards unsettled directions, and GFP fluorescence of MTs connecting two poles was extremely weak (Fig. 1A).

Behaviour and function of Peg1 have been investigated particularly regarding those in anaphase. The function of Peg1 before anaphase, however, has not been clarified yet. We therefore observed spindle assembly in the *peg1-104* mutant using live-cell imaging.

In general, kinetics of the spindle length (inter-SPB distance) in WT cells can be sectioned into three phases: the spindle starts to grow in prophase, which is hallmarked by SPB separation (0–6 min, WT; Fig. 1B). This state, called ‘phase 1’, lasts for 2–3 min (Nabeshima et al., 1998). The spindle then reaches a constant length (∼2 μm) without elongation for more than 5 min, which is called ‘phase 2’ (6 min, WT) corresponding to prometaphase to metaphase. The spindle resumes to elongate in anaphase (‘phase 3’; 14 min, WT). The three-phase development of the spindle was precisely reproduced in our WT strains (Fig. 1C).

In contrast, *peg1-104* cells exhibited severe defects in spindle formation: SPBs did not fully separate in phase 1, and the spindle size did not reach 2 μm unlike in WT cells. Most of *peg1-104* cells we observed were unable to start anaphase spindle elongation (Fig. 1C). This phenotype seen in the *peg1-104* mutant is similar to phenotypes of other *peg1* mutant alleles (*peg1.1*, containing the L747F mutation; and *cls1-36*, containing I739T, D804V, E946K and I1277M mutations) (Bratman and Chang, 2007; Grallert et al., 2006). The spindle defects in pre-anaphase could be explained by MT bundling defects as seen in Fig. 1A. This was then investigated using live-cell imaging with higher time resolution. During WT pre-anaphase, most MTs nucleated from SPBs were bundled towards the spindle axis to build up the pole-to-pole spindle, therefore only few MTs remained unbundled (1 min 46 s, WT; Fig. 1D). In the *peg1-104* mutant, however, unbundled MTs emanating from SPBs were constantly observed (Fig. 1D), reflecting that MTs were frequently unbundled and instead extended outwards off the spindle axis (seen in 22% of observed mitotic WT cells; 100% of *peg1-104* cells; Fig. 1E).

When the spindle elongates in WT anaphase (phase 3), Peg1 is shown to be loaded onto the MT interdigitating zone in the middle of the spindle, through association with the antiparallel MT bundling factor Ase1, in order to stabilise the spindle midzone (Bratman and Chang, 2007; Loïodice et al., 2005; Yamashita et al., 2005). By looking at Peg1 behaviours in anaphase, Peg1 in pre-anaphase might be also dependent upon Ase1 in order to bundle MTs. To test this possibility, we performed similar MT observations using *ase1*Δ (complete deletion) mutant strains. As reported previously (Loïodice et al., 2005; Yamashita et al., 2005), *ase1*Δ cells frequently showed spindle collapse, particularly during anaphase spindle elongation (16 min, *ase1*Δ; Fig. 1B). Such collapse was not evident during pre-anaphase (phase 2); most of *ase1*Δ cells were possible to transit from phase 2 to phase 3, indicating a sharp contrast to *peg1-104* cells, which did not enter phase 3 (Fig. 1C).

In line with this, *ase1*Δ cells did not frequently show unbundled MTs in time-lapse imaging (Fig. 1D,E). These results suggest that Ase1 does not play essential functions for spindle MT bundling during pre-anaphase, although it is required for MT bundling in later stages; namely for anaphase spindle elongation (Loïodice et al., 2005; Yamashita et al., 2005). These results indicate that Peg1 may function without Ase1 for MT bundling during pre-anaphase, unlike in anaphase.

To quantify how frequently unbundling MTs are generated in the *peg1-104* mutant, we next visualised Mal3/EB1, which decorates the plus ends of growing MTs (Beinhauer et al., 1997; Busch and Brunner, 2004). In WT cells, Mal3-GFP localised to MTs almost exclusively in between two SPBs (Fig. 1F). In *peg1-104* cells, on the contrary, Mal3-GFP scattered around the spindle axis, which is indicative of unbundled MTs. Each unbundled MT appeared to dynamically change the growth direction (Fig. 1F). The frequency of unbundled MTs bearing Mal3-GFP appeared per minute was elevated in the *peg1-104* mutant (1.4 MTs per min in WT; 13.0 MTs per min in *peg1-104*, Fig. 1G), reconfirming that *peg1-104* cells were defective in MT bundling in the pre-anaphase spindle. Thus, the phenotype was reproduced when Mal3-GFP was visualised instead of GFP-Atb2. We therefore excluded the possibility that the unbundled MT phenotype was due to artificial tagging of α-tubulin with GFP.

Taken together, we revealed a function of Peg1 to bundle MTs in the pre-anaphase spindle, which is distinct from the known function of Peg1 in anaphase assisted by Ase1.

### MT dynamics was not affected by the *peg1-104* mutation

It has been reported how Peg1 changes MT dynamics (Bratman and Chang, 2007; Grallert et al., 2006). An *in vitro* study reported that the growth rate and the rescue frequency increased by the recombinant Peg1 protein, whereas the shrinkage rate and the catastrophe frequency decreased (Al-Bassam et al., 2010). MT dynamics *in vivo* have been measured using interphase *Schizosaccharomyces* *pombe* cells: Grallert et al. (2006) showed that the growth rate slightly increased but the shrinkage rate and the catastrophe frequency decreased in the *peg1.1* mutant. Bratman and Chang (2007), on the other hand, showed that growth and shrinkage rates and the catastrophe frequency was comparable in WT and in *cls1-36/peg1* mutant cells. They also suggested that Peg1 may promote rescue events: conversion from shrinkage to growth, in late mitosis.

**Fig. 2.**
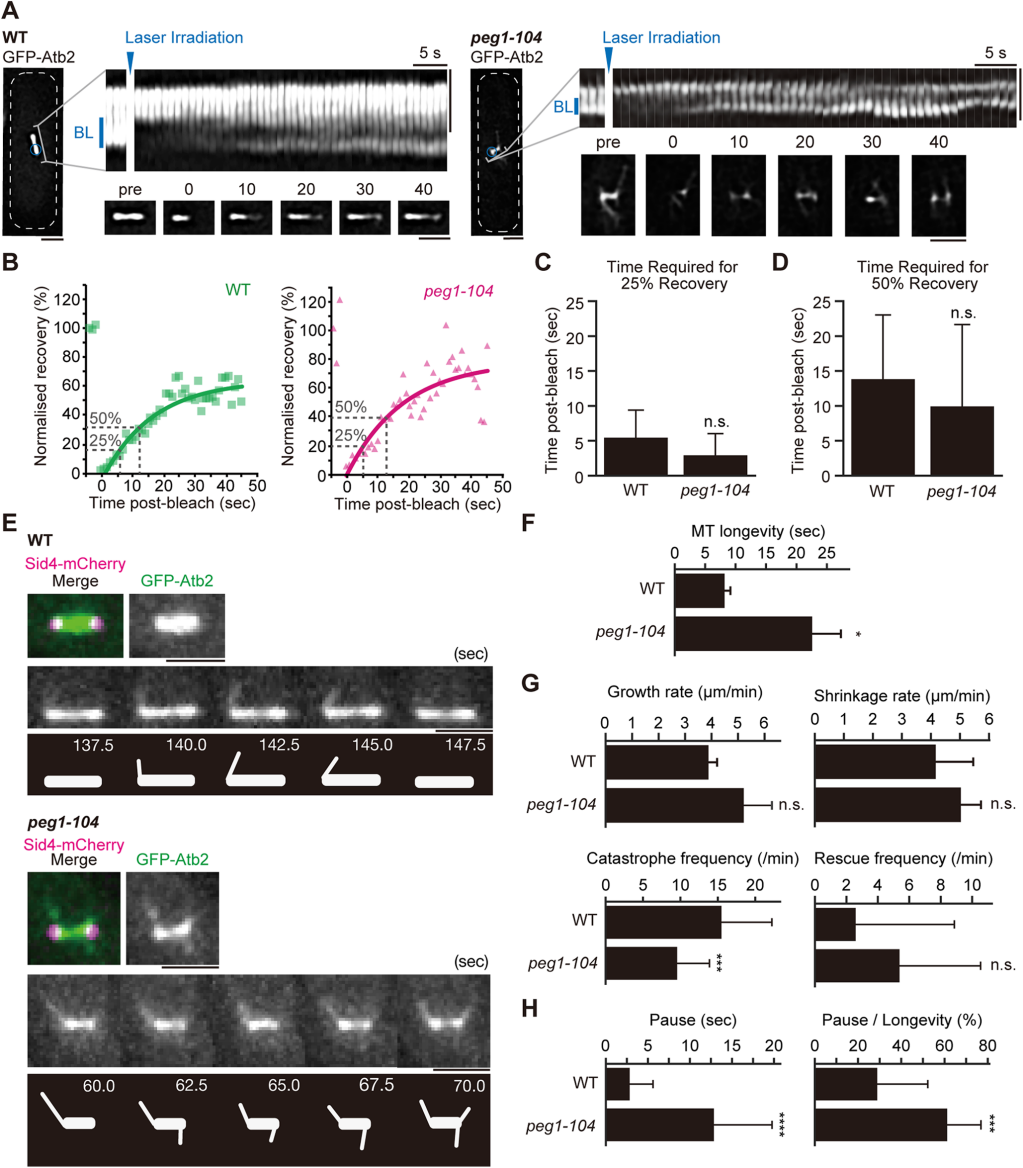
**Dynamic properties of spindle MTs in *peg1-104*.** (A–D) Fluorescence recovery after photobleaching (FRAP) assays were done using WT and *peg1-104* cells expressing GFP-Atb2. A half of the pre-anaphase spindle (inset, A) was photobleached by laser irradiation at 0 s (arrowheads), and time-lapse images before (pre) and after bleach were filmed every second and shown as kymograph. Cell shape and a bleached region are outlined as a white dotted line and a blue circle, respectively. ‘BL’ represents the bleached region. Kinetics of fluorescence recovery is shown (B). Fluorescence intensity of GFP-Atb2 in the bleached region normalised using background signal was plotted at each timepoint and fitting curves are drawn. Right, WT (green squares); left, *peg1-104* (magenta triangles). Time required for 25% recovery (C) and for 50% recovery (D) are indicated in each graph. Error bars, s.d. *n*=16–18, WT; *n*=17–19, *peg1-104*. (E–H) Dynamic properties of unbundled MTs in WT and *peg1-104* spindles. Pre-anaphase spindles were filmed every 2.5 s at 32°C (E). GFP-Atb2 images are shown with schematics illustrating behaviour of unbundled MTs. For unbundled MTs observed in E, the following dynamic properties were measured: longevity (F), rates for growth and shrinkage, frequencies of catastrophe and rescue (G), length of pause and the ratio of pause to MT longevity (H). *N*=3 experiments, *n*=3–23 MTs, WT; *n*=5∼33 MTs, *peg1-104*. Error bars, s.d.; *P<0.05, ***P<0.0005, ****P<0.00005; n.s., no significance; two-tailed Student's *t*-test, except for catastrophe frequency (G) and pause (H) (two-tailed Welch's *t*-test). Scale bars: 2 μm.

Nothing has been reported to date regarding effects of Peg1 on MT dynamics in pre-anaphase. To examine effects of the *peg1-104* mutation on MTs in the pre-anaphase spindle, we performed fluorescence recovery after photobleaching (FRAP) assays. Half of the pre-anaphase spindle in WT and *peg1-104* cells expressing GFP-Atb2 were photobleached by irradiating 488 nm laser, followed by capturing GFP-Atb2 images every second to chase temporal kinetics of fluorescence recovery (Fig. 2A). Both spindles in WT and *peg1-104* cells recovered fluorescence intensity of GFP-Atb2 around the spindle pole several seconds after bleaching (Fig. 2A). The rate for fluorescence recovery in *peg1-104* cells was comparable to that in WT cells (Fig. 2B). Time required for 25% recovery (Fig. 2C) and for 50% recovery (Fig. 2D) of the mobile GFP-Atb2 fraction did not show significant difference between WT and *peg1-104* cells. These results suggest that Peg1 does not affect overall assembly of spindle MTs in pre-anaphase.

We next assessed detailed dynamics of spindle MTs. The *S. pombe* spindle displays linear morphology with dense MTs, which hampered analyses of dynamics of each MT bundle therein. We then focused on unbundled MTs that were located off the main body of the spindle (Fig. 2E). First, the lifespan of unbundled MTs was longer in the *peg1-104* than in WT (*P*=0.014; Fig. 2F). Comparison between them indicated no statistical significance in the growth rate (*P*=0.165, two-tailed Student's *t*-test; Fig. 2G), as well as in the shrinkage rate (*P*=0.451; Fig. 2G). The catastrophe frequency of unbundled MTs was slightly lower in *peg1-104* than in WT cells (*P*=0.00024; Fig. 2G), whereas the rescue frequency was similar to each other (*P*=0.095; Fig. 2G). We also monitored the ‘pause’ state of unbundled MTs. ‘Pause’ was defined when MT length changed less than 0.2 µm. The duration of MT pause was longer in *peg1-104* cells, and the percentage of the MT pause state during the MT lifespan was higher in the mutant than in WT (Fig. 2H). Taken together, we conclude that Peg1 in pre-anaphase bundles MTs, as well as modulating dynamics of MTs before bundling.

### Peg1 is required for proper segregation of chromosomes

**Fig. 3.**
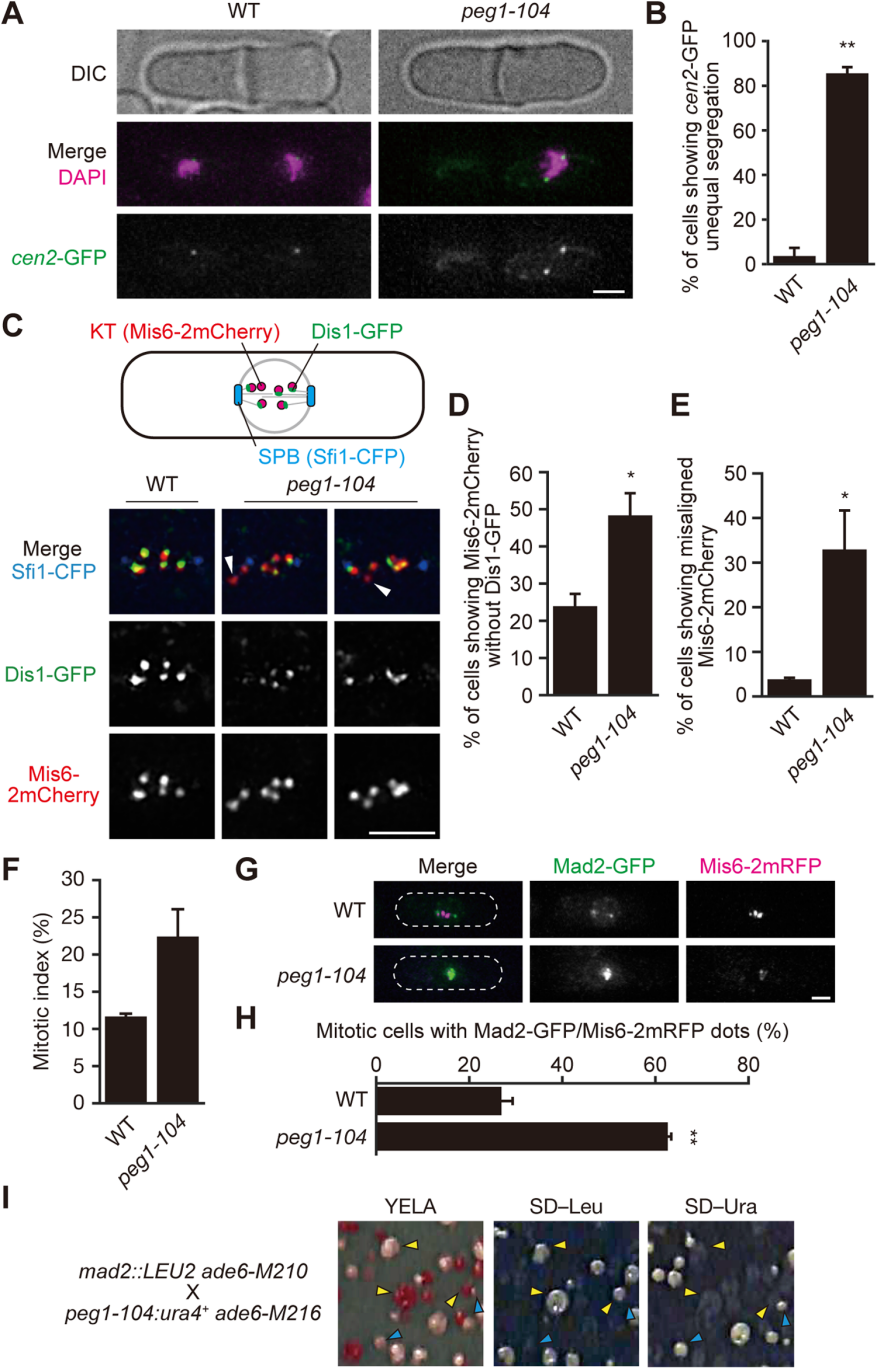
**Peg1 ensures kinetochore-microtubule attachment.** (A,B) Chromosomes were unequally segregated in the *peg1-104* mutant. *cen2*-GFP (a marker for the centromere of chromosome II, green) and DAPI (magenta) are shown in post-mitotic WT and the *peg1-104* cells (A). Percentages of unequal segregation of *cen2*-GFP are shown (B). *N*=2 experiments. *n*=41, 43, WT; *n*=43, 52, *peg1-104*. (C–E) Kinetochores lacking Dis1 were frequently seen in the *peg1-104* mutant. Dis1-GFP (green) was visualised with Mis6-2mCherry (red) and Sfi1-CFP (blue) in WT and *peg1-104* cells at 32°C (C). A schematic for filmed images is shown on the top. Nuclear regions are shown enlarged (bottom). Absence of Dis1-GFP on some kinetochores was seen in the *peg1-104* mutant (arrowheads). Frequencies of cells showing Mis6-2mCherry without Dis1-GFP (D) and showing misaligned Mis6-2mCherry (E). *N*=3 experiments, *n*=23–28, WT; *n*=25–30, *peg1-104.* Error bars, s.d. (F) Mitotic index. Error bars, s.d. *N*=2 experiments, *n*=166, 301, WT; *n*=203, 331, *peg1-104*. (G,H) WT and *peg1-104* cells expressing Mad2-GFP (green) and Mis6-2mRFP (kinetochores, magenta) grown at 32°C were fixed and observed. Cell shape is outlined with dotted lines. Frequencies of mitotic cells displaying Mad2-GFP foci co-localised with Mis6-2mRFP are also shown (H). *N*=2 experiments, *n*=49, 58, WT; *n*=101, 147, *peg1-104*. (I) *peg1-104* is synthetic lethal with *mad2*Δ. Offspring after genetic crossing of two indicated strains were replica-plated onto indicated media. Colonies grown on SD medium without leucine (SD–Leu) contain the *mad2*Δ mutation marked by the *LEU2* gene (yellow arrowheads). Colonies grown on SD–Ura contains the *peg1-104* mutation marked by the *ura4*^+^ gene (blue arrowheads). No colonies grew on both of the media. Error bars, s.d.; *P<0.05, **P<0.005; two-tailed Student's *t*-test, except for E (two-tailed Welch's *t*-test). Scale bars: 2 μm.

How do the defects in MT bundling in pre-anaphase affect progression of mitosis? We first assessed if chromosome segregation was defective in the *peg1-104* mutant, in which centromeres of chromosome 2 were labelled by GFP (the *cen2*-GFP system; Yamamoto and Hiraoka, 2003) stained with DAPI (4′,6-diamidino-2-phenylindole). We found that ∼85% of *peg1-104* cells displayed unequal segregation of the *cen2*-GFP signal (Fig. 3A,B). Although the DAPI signal was not split into two parts, two separated *cen2*-GFP dots were seen in *peg1-104* cells (Fig. 3A). We then suspected that *peg1-104* cells could not enter anaphase A (the stage for chromosome segregation).

To test this possibility, we filmed metaphase *peg1-104* cells (*n*=33). ∼85% of *peg1-104* cell (28/33) failed in *cen2*-GFP separation before cytokinesis (right, Fig. S3). Although ∼15% of *peg1-104* cells (5/33) separated *cen2*-GFP dots into two, those dots remained in a single compartment after cytokinesis (left, Fig. S3). These results suggest that *peg1-104* cells displayed severe defects in segregation of sister chromatids in anaphase A, because of bundling defects of spindle MTs accumulated until metaphase.

### Peg1 promotes kinetochore-microtubule attachment

Failure in chromosome segregation in the *peg1-104* mutant may be due to defects in attachment of MTs to kinetochores (KTs). We therefore observed localisation of the conserved MAP Dis1/XMAP215 (Ohkura et al., 1988) as a marker for KT-MT attachment, as Dis1 appears delivered by spindle MTs to KTs (Hirai et al., 2014). Forty-eight percent of *peg1-104* pre-anaphase cells had kinetochores that did not accompany Dis1-GFP, whereas only ∼24% of WT cells did (Fig. 3C,D). In line with this, misaligned chromosomes (without Dis1-GFP) that were positioned apart from the spindle were seen in ∼33% of *peg1-104* cells, in comparison with only 3.8% of WT cells (white arrowheads, Fig. 3C,E). These results indicate that KT-MT attachment is defective in the *peg1-104* mutant.

We next focused on spindle assembly checkpoint, which monitors unattached KTs during pre-anaphase (for review, see Foley and Kapoor, 2013). First, in asynchronous cultures, frequencies of mitotic cells (mitotic index) was ∼12% in WT cells and ∼22% in *peg1-104* cells (Fig. 3F), suggesting a mitotic delay possibly caused by the checkpoint machinery. The frequency of mitotic cells showing Mad2-GFP dots at KTs (Fig. 3G) was 63% in *peg1-104*, compared to 27% in WT cells (Fig. 3H), indicating that the checkpoint is frequently activated in *peg1-104* cells. Genetic crossing between *peg1-104* and *mad2*Δ strains turned out to be synthetic lethal (Fig. 3I), showing that the spindle assembly checkpoint is vital for survival of the *peg1-104* mutant.

Mammalian CLASPs are shown to localise to KTs in mitosis (Maiato et al., 2003; Pereira et al., 2006). A previous study reported that Peg1 colocalised with the kinetochore component Ndc80 when cells were arrested at metaphase using the β-tubulin mutant *nda3-311* (Bratman and Chang, 2007). We therefore re-examined whether Peg1 localises to KTs to promote KT-MT attachment. Peg1-3GFP in fixed cells, however, did not localise to KTs at any time during the cell cycle (Fig. S4). Taken together, Peg1 plays a role in MT bundling particularly before anaphase, whose function is essential for establishment of KT-MT attachment.

### Peg1 localises to the pre-anaphase spindle independently of Ase1

In later stages of mitosis, Peg1 is shown to be recruited to the anaphase spindle midzone through interaction with Ase1, a well-defined MT bundling factor, thereby stabilising MTs (Bratman and Chang, 2007).

**Fig. 4.**
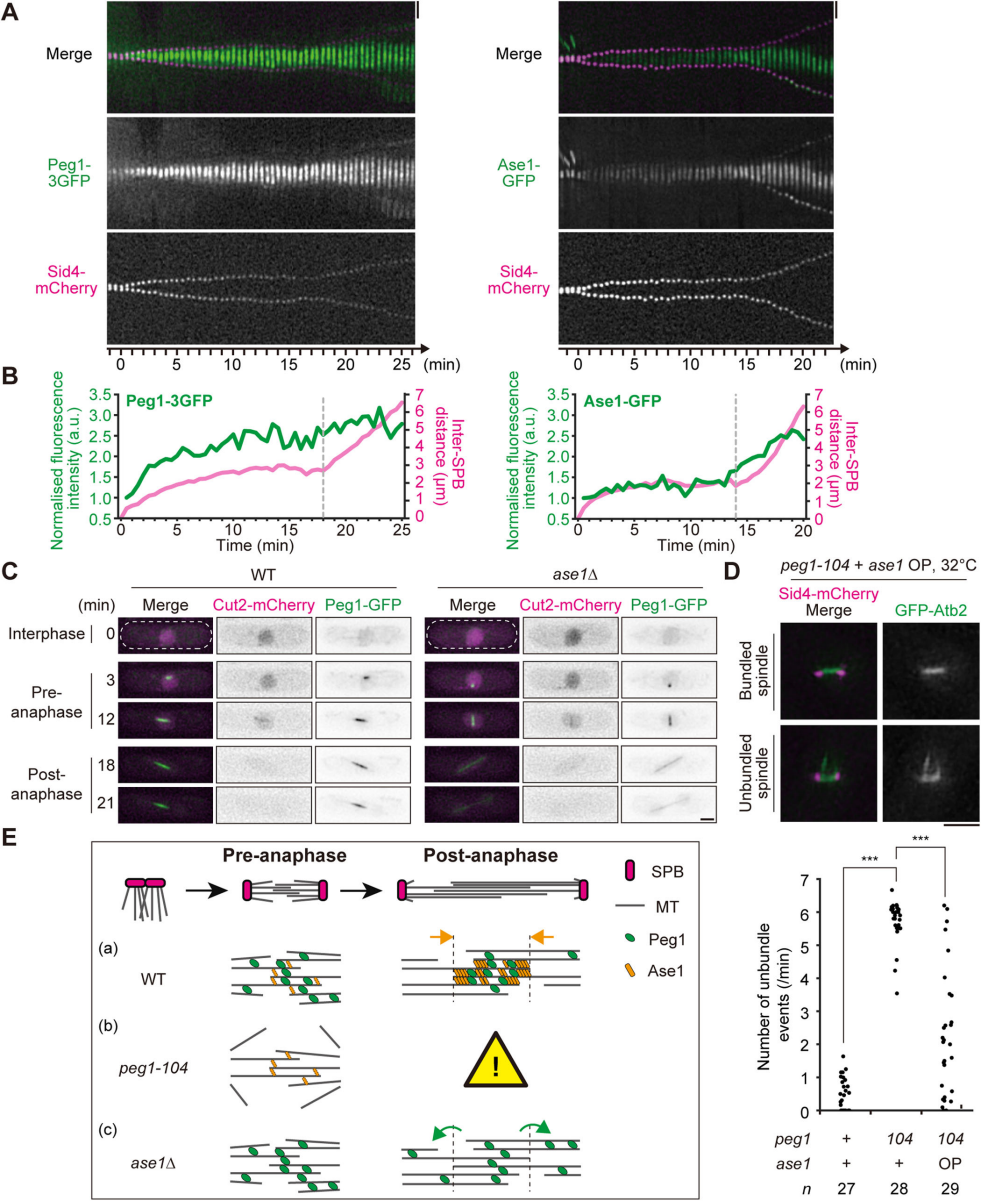
**Peg1 localises to the pre-anaphase spindle in an Ase1-independent manner.** (A,B) Localisation of Peg1-3GFP and Ase1-GFP (green) to the spindle was observed with Sid4-mCherry (magenta) every 30 s throughout mitosis, and kymographs are shown (A). Kinetics of the Inter-SPB distance and total fluorescence intensity of Peg1-3GFP or Ase1-GFP on the spindle (B). The intensity at each timepoint was normalised by the one at 0.5 min. 0 min corresponds to the onset of SPB separation. Dotted lines in B provide the timing of phase II–III transition. (C) Peg1 localises to the spindle before anaphase in an Ase1-independent manner. Peg1-GFP (green) was visualised with Cut2-mCherry (magenta) in WT and *ase1*Δ cells. Cell shape is outlined with dotted lines. (D) Overproduction of Ase1 partially suppressed unbundled MTs in *peg1-104* cells. MTs were visualised with GFP-Atb2 (green) and Sid4-mCherry (magenta) every 10 s and frequencies of unbundle events are shown in the graph. ***P<0.0005, two-tailed Student's *t*-test between ‘*peg1*^+^ *ase1*^+^’ and ‘*peg1-104 ase1*^+^’, two-side Welch's *t*-test between ‘*peg1-104 ase1*^+^’ and ‘*peg1-104 ase1* OP’. (E) A model for MT bundling in mitosis. Scale bars: 2 μm.

On the other hand, the role of Peg1 in MT bundling in pre-anaphase may not depend upon Ase1, as *peg1-104* and *ase1*Δ mutants exhibited distinct phenotypes (see Fig. 1). This is also supported by previous notion that Ase1 (PRC1) is phosphorylated by Cdc2/Cdk1 (cyclin dependent kinase) in pre-anaphase, which prevents Ase1 localising to the spindle midzone (Fu et al., 2009; Mollinari et al., 2002). Indeed, Ase1-GFP showed midzone-specific localisation predominantly during anaphase spindle elongation (Loïodice et al., 2005; Yamashita et al., 2005), whereas Peg1-3GFP localised to the spindle even from pre-anaphase (Fig. 4A,B). In prophase right after SPBs started to separate, Peg1-3GFP localised to the almost entire spindle (0.5–3 min, Fig. 4A), which became gradually restricted to the midzone (3–25 min, Fig. 4A).

Thus, it is during anaphase that Peg1 and Ase1 share similar localisation to the spindle midzone, and Peg1 in pre-anaphase does not appear to require Ase1 localisation to the midzone. To test the hypothesis, Peg1 localisation was observed in *ase1*Δ cells. Unlike in the previous study, we were unable to create the strain expressing Peg1-GFP instead of endogenous (untagged) Peg1 in the *ase1*Δ background (Bratman and Chang, 2007). We then had to adopt the strain expressing Peg1-GFP ectopically from another gene locus termed *co2* (Kakui et al., 2015) in addition to the endogenous untagged Peg1, in the *ase1*Δ background. As previously reported, the midzone localisation of Peg1-GFP was lost in *ase1*Δ, confirming that accumulation of Peg1 to the spindle midzone in anaphase requires Ase1 (Fig. 4C) (Bratman and Chang, 2007), although Peg1 could weakly associate to the spindle independently of Ase1. On the other hand, we found that Peg1-GFP localised properly to the pre-anaphase spindle even in *ase1*Δ cells (Fig. 4C). We thus concluded that localisation of Peg1 is regulated differentially in early and in late mitosis: namely, Peg1 localises to the spindle in an Ase1-independent manner in early mitosis, whereas in late mitosis it localises in an Ase1-dependent manner.

### Peg1 is a MT bundling factor in pre-anaphase

Those results led us to postulate that it is Peg1 that directly bundles spindle MTs during pre-anaphase, although Ase1 bundles antiparallel MTs in anaphase. To further clarify the bundling function of Peg1, Ase1 was ectopically overproduced in *peg1-104* cells to test its effects on unbundled MTs during pre-anaphase. As shown in Fig. 4D, all of observed *peg1-104* cells showed unbundled MTs during pre-anaphase. In contrast, when Ase1 was overproduced in *peg1-104* cells, the frequency of cells displaying unbundled MTs decreased significantly (Fig. 4D). These results demonstrate that the MT-bundling factor Ase1 could substitute function of Peg1 during pre-anaphase spindle organisation, when artificially utilised.

## DISCUSSION

In summary, we propose that Peg1/CLASP promotes MT bundling during pre-anaphase to assemble the bipolar spindle. As modelled in Fig. 4E (a), Peg1 localises to the spindle throughout mitosis, but its localisation pattern and function are regulated differentially depending upon the stage during mitosis.

Prior to anaphase, Peg1 localises to the entire spindle to bundle MTs [Fig. 4E (a)]. This function of Peg1 is essential, as the *peg1-104* mutant of the stage showed severe defects in MT bundling, which hampered further development of the spindle in late mitosis [Fig. 4E (b)]. Further studies such as *in vitro* assays and electron microscopy would strengthen our model proposing Peg1 as an intrinsic bundling factor.

The *peg1-104* mutant also resulted in frequent unequal segregation of chromosomes, clearly demonstrating that the bundling function of Peg1 is required before anaphase, meaning that the function is separable from the Peg1 function in anaphase spindle elongation published previously (Bratman and Chang, 2007). Involvement of other CLASP orthologues in chromosome segregation have been reported for various organisms. *Drosophila* MAST/Orbit is required for polymerisation of kinetochore-mediated MTs (Maiato et al., 2005). *Xenopus* Xorbit is required for chromosome congression and bipolar spindle formation during metaphase (Hannak and Heald, 2006). Mammalian CLASP1 and CLASP2 localise to kinetochores and modulate spindle MT dynamics (Maiato et al., 2003; Mimori-Kiyosue et al., 2006; Pereira et al., 2006). In general, CLASPs in those organisms modulate MT dynamics, but we propose a new function for CLASPs: bundling of MTs in the pre-anaphase spindle, which contributes to sister chromatid separation.

After the onset of anaphase in WT, Ase1 intensively accumulates to the MT overlapping zone at the midzone of the spindle (Loïodice et al., 2005; Yamashita et al., 2005), which physically recruits Peg1 to restrict its localisation predominantly to the midzone to stabilise MTs [Fig. 4E (a)] (Bratman and Chang, 2007). This translocation of Peg1 indeed depends upon Ase1, as Peg1 dispersed uniformly to the entire spindle when Ase1 was depleted [Fig. 4E (c)].

On the other hand, the MT bundling function of Peg1 before anaphase did not rely on Ase1, because *ase1*Δ cells still maintained both Peg1 localisation and bundled MTs in pre-anaphase [Fig. 4E (c)]. Our results thus demonstrate that both Peg1 and Ase1 function as MT bundling factors, although the timing at which each factor operates is distinct: namely, Peg1 before anaphase and mainly Ase1 (supported by Peg1 as a MT stabiliser) in anaphase.

Before anaphase, Cdk/Cdc2 phosphorylates Ase1 to block its spindle localisation (Fu et al., 2009), which allows Peg1 to bundle parallel MTs. It is possible that spindle assembly at this stage requires Peg1, which bundles parallel MTs, but excludes Ase1, which bundles anti-parallel MTs. In anaphase, dephosphorylated Ase1 accumulates to and bundles interdigitating MTs and recruit Peg1 there, suggesting that both parallel and anti-parallel bundling are required to maintain the integrity of the anaphase spindle at the midzone. Thus, the cyclin-dependent kinase plays a pivotal role in switching the MT-bundling system operated by the synergistic interplay of Peg1 and Ase1 at anaphase onset.

## MATERIALS AND METHODS

### Yeast strains and genetics

Strains used in this study are listed in Table S1. Standard methods for yeast genetics and gene tagging were used (Moreno et al., 1991; Sato et al., 2005). YE5S (the YE medium containing five supplements) was used for growing *S. pombe* strains unless otherwise stated. The methods for tagging tandem copies of fluorescent proteins were described previously (Sato et al., 2009). For visualisation of microtubules, *Z2-GFP-atb2-kanR* strains were used, in which the *GFP-atb2* (α-tubulin) fusion gene driven by the native *atb2*^+^ promoter was inserted in the Z2 region of a chromosome, to express GFP-Atb2 in addition to endogenous Atb2 proteins (Ohta et al., 2012).

Genetic crossing was performed by placing cell suspension of two parental strains onto SPA (sporulation agar) plates to induce mating, meiosis and sporulation. Spores were then placed on YE5S plates, and colonies were replica-plated onto the indicated selection plates to identify the genotype of each offspring colony.

The strain overproducing Ase1 (HE11) in Fig. 4D was constructed as follows: using PCR amplification, DNA fragments containing the nourseothricin (clonNAT; WERNER BiogAgents, Germany) resistance gene as a selection marker and the *nmt1*^+^ promoter (*nat-Pnmt1*) were flanked by targeting sequences for the 5′-end of the *ase1*^+^ gene. Transformants by the fragment were selected on YE5S plates containing nourseothricin, and then correct integration of the fragment was confirmed through colony-PCR assays.

For ectopic expression of Peg1-mCherry (Fig. S2D), The fragment *Ppeg1-peg1-mCherry-T_adh_-bsd* (the promoter and coding sequences of the *peg1* gene fused with the *mCherry* gene and marked by the *bsd* selection marker gene) was inserted at the *co2* locus on chromosome I through transformation (Kakui et al., 2015). The resultant was double-checked by assays using blasticidin S (FUJIFILM Wako Pure Chemical, Japan) and by colony-PCR.

### Microscopy

Cells except in Fig. 4D were grown in YE5S at 25°C (Fig. 4A,C; Fig. S2A). For observation of temperature-sensitive mutants, strains were shifted to 32°C right before observation (Figs 1D and 2A), or 3 h before observation (Figs 1A,B,F, 2E and 3; Figs S3 and S4). For Fig. 4D, indicated strains were grown in EMM+N (Edinburgh minimal medium with the nitrogen source), containing supplements and 2 μg ml^−1^ thiamine (Sigma-Aldrich) at 25°C for 19 h, and then shifted to 32°C for 3 h prior to observation.

Live-cell imaging was performed with the DeltaVision-SoftWoRx system (Applied Precision) equipped to the Olympus IX71/81 microscope as previously described (Sato et al., 2009). Briefly, cells were mounted on a 35-mm glass-bottomed dish (Matsunami, Japan) coated with lectin (Sigma-Aldrich), which was then filled with EMM+N with supplements. Images were acquired as serial sections along the z-axis and stacked using the ‘quick-projection’ algorithm in SoftWoRx. The captured images were processed using Photoshop CC 2019 (Adobe, San Jose, CA, USA) or Fiji (Schindelin et al., 2012). Contrast of images shown in Fig. 3C (Dis1-GFP) and Fig. 3G (Mis6-2mRFP) were adjusted using the software. For Fig. 3 and Fig. S4, indicated strains were fixed with 3.2% formaldehyde (Thermo Fisher Scientific) for 15 min, and mounted on a slide glass (Matsunami, Japan). For DAPI (4′,6-diamidino-2-phenylindole) staining of fixed cells in Fig. 3A, VECTASHIELD Mounting Medium with DAPI (Vector Laboratories, Burlingame, CA, USA) was used.

For Fig. 2A–D, fluorescence recovery after photobleaching (FRAP) experiments were done as previously described (Sato et al., 2009) with slight modifications. Briefly, the QLM-laser bleaching system (Seki technotron) equipped to the DeltaVision-SoftWoRx system was used. A circular region with an average radius of 0.5–1 μm including the GFP-Atb2 signal in half of the spindle was photobleached with the 488-nm laser (power: 50%, duration: 0.8 s). Three images were taken before bleaching as a reference, followed by 46 images after bleaching at 1-s intervals. For data analysis, mean fluorescence intensity of the bleached region (*F_bl*), a non-bleached reference region (*F_ref*) and a background region (only noise; *F_bgd*) were measured with Fiji. After subtraction of the background noise, the fluorescence intensity in the bleached region was normalised by the reference region in 49 images [*F_bl_norm*=(*F_bl* – *F_bgd*)/(*F_ref* – *F_bgd*)]. *F_bl_norm* was finally normalised by an average value of three pre-bleach timepoints. Curve fitting in Fig. 2B was operated in Fiji using the ‘Exponential Recovery’ model. Parameters of formulae describing the fitted curve were used to determine the time required for 25% (Fig. 2C) and 50% recovery (Fig. 2D).

To measure microtubule dynamics (Fig. 2E–H), coordinates corresponding positions of both ends of unbundled MTs were recorded from images of each timepoint and the length of unbundle MTs was calculated. Longevity of unbundled MTs (Fig. 2F) was defined as the time an unbundled MT lasted. Growth rate, shrinkage rate, catastrophe frequency and rescue frequency (Fig. 2G) were calculated using the values. ‘Pause’ state was defined when MT length changed less than 0.2 µm. ‘Pause’ time and its ratio to the longevity (Fig. 2H) were calculated using the values.

### Protein analyses

Proteins were extracted from cells using standard procedures as follows: for Fig. S2B,C, cells were cultured in YE5S at 25°C overnight, and a portion of the culture was collected for protein extraction. The rest of the culture was shifted to 32°C for 3 h and then collected. Cells were washed with STOP buffer (150 mM NaCl, 50 mM NaF, 10 mM EDTA [pH 8.0], 1 mM NaN_3_). After centrifugation, cell pellets were suspended in HB buffer [25 mM MOPS pH 7.2, 15 mM MgCl_2_, 5 mM EGTA, 0.1 mM Sodium Vanadate, 150 mM KCl, 50 mM β-Glycerophosphate, 15 mM *p*-nitrophenylphosphate, 0.2% NP-40, 1 mM dithiothreitol, 1 mM phenylmethanesulfonyl fluoride, Complete protease inhibitor cocktail (Roche)] and then lysed with glass beads in a FastPrep-24 beads shocker (MP Biomedicals; 25 s three to four times, power 5.5) at 4°C. After centrifugation at 1500×***g*** for 1 min followed by additional two to three rounds of centrifugation at 5800×***g*** for 2 min at 4°C, the supernatants were collected as protein extracts. For immunoblotting, SNAP i.d. 2.0 (Millipore) was used. Each membrane was soaked in 5% skim milk for 1 h and 0.1% skim milk for 15 min. The skim milk solution was prepared in phosphate-buffered saline containing 0.1% Tween 20 (Kanto Chemical, Japan). Membranes were then blotted with the anti-GFP antibody (7.1 and 13.1 mixture) (1:2500, Roche) or with the anti-α-tubulin (B-5-1-2) antibody (1:3000, Santa Cruz Biotechnology) as the primary antibody. Horseradish peroxidase-conjugated anti-mouse IgG (1:2500; Jackson ImmunoResearch Laboratories) was used as the secondary antibody. The band intensity was quantified using Fiji. After subtracting the background intensity, ratios of intensities for anti-GFP to anti-α-tubulin were calculated for comparison (Fig. S2C).

## Supplementary Material

Supplementary information
