# Supplementary material for: CLASP promotes microtubule bundling in metaphase spindle independently of Ase1/PRC1 in fission yeast
Source: Biol Open. 2019 Oct 15;8(10):bio045716. doi: 10.1242/bio.045716 (PMC6826280; doi:10.1242/bio.045716)
Supplement: Supplementary information [file biolopen-8-045716-s1.pdf]

Table S1: ***S. pombe*** **strains used in this study**

Strain	Genotype	Figure
JY878	*h90 ade6-M216 leu1 ura4*	S2B-D
MJ260	*h- mad2-GFP-kanR mis6-2mRFP-hph sfi1-CFP-natR ade6-M210 leu1 ura4*	3F-H
KRY128	*h90 cen2<<lacO-kanR-ura4+ his7+<<(dis1pro)-GFP-lacI sfi1-CFP-natR ade6-M216 leu1 ura4 his2*	3A,B
LJ5	*h90 peg1-GFP-kanR ade6-M216 leu1 ura4*	S2B,C
LJ27	*h90 peg1-3GFP-kanR mis6-2mRFP-hph sfi1-CFP-natR ade6-M216 leu1 ura4*	S4
LJ523	*h90 dis1-GFP-kanR mis6-2mCherry-hph sfi1-CFP-natR ade6-M210 leu1 ura4*	3C-E
LJ525	*h90 peg1-104:ura4+ ade6-M216 leu1 ura4*	S2D
LJ530	*h90 peg1-104-GFP-kanR ade6-M216 leu1 ura4*	S2B,C
LJ591	*h90 peg1-104 mal3-GFP-kanR sid4-mCherry-hph ade6-M216 leu1 ura4*	1F,G
LJ592	*h90 mal3-GFP-kanR sid4-mCherry-hph ade6-M216 leu1 ura4*	1F,G
LJ600	*h90 sid4-mCherry-hph ade6-M216 leu1 ura4*	S2A
LJ634	*h90 Z2-GFP-atb2-kanR sid4-mCherry-hph ade6-M216 leu1 ura4*	1A-E,2,4D
LJ635	*h90 peg1-104 Z2-GFP-atb2-kanR sid4-mCherry-hph ade6-M216 leu1 ura4*	1A-E,2,4D
LJ649	*h90 peg1-GFP-kanR sid4-mCherry-hph ade6-M216 leu1 ura4*	S2A
LJ650	*h90 peg1-104-GFP-kanR sid4-mCherry-hph ade6-M216 leu1 ura4*	S2A
LJ658	*h90 ase1::ura4+ Z2-GFP-atb2-kanR sid4-mCherry-hph ade6-M216 leu1 ura4*	1B-E
LJ666	*h90 ase1-GFP-kanR sid4-mCherry-hph ade6-M216 leu1 ura4*	4A,B
HE11	*h90 peg1-104 natR-Pnmt1-ase1 Z2-GFP-atb2-kanR sid4-mCherry-hph ade6-M216 leu1 ura4*	4D
HE123	*h90 peg1-104-ura4+ co2::Ppeg1-peg1-mCherry-bsdR ade6-M216 leu1 ura4*	S2D

Biology Open • Supplementary information

HE284	*h- peg1-104 mad2-GFP-kanR mis6-2mRFP-hph sfi1-CFP-natR ade6-M210 leu1 ura4*	3F-H
HE287	*h- peg1-104:ura4+ mis6-2mRFP-hph sfi1-CFP-natR ade6-M216 leu1 ura4*	3I
HE369	*h90 peg1-3GFP-kanR sid4-mCherry-hph ade6-M216 leu1 ura4*	4A,B
HE376	*h90 peg1-104-ura4+ cen2<<lacO-kanR-ura4+ his7+<<(dis1pro)-GFP-lacI Z2-mCherry-atb2-hph sfi1-CFP-natR ade6-M? leu1 ura4*	S3
HE381	*h+ mad2::LEU2 ade6-M210 leu1 ura4 his2*	3I
HE462	*h90 cut2-mCherry-hph co2::Ppeg1-peg1-GFP-bsdR ade6-M216 leu1 ura4*	4C
HE465	*h90 cut2-mCherry-hph ase1::ura4+ co2::Ppeg1-peg1-GFP-bsdR ade6-M216 leu1 ura4*	4C
HE499	*h90 peg1-104 dis1-GFP-kanR mis6-2mCherry-hph sfi1-CFP-natR ade6-M? leu1 ura4*	3C-E
HE503	*h90 peg1-104 cen2<<lacO-kanR-ura4+ his7+<<(dis1pro)-GFP-lacI sfi1-CFP-natR ade6-M216 leu1 ura4 his2*	3A,B

The original strain for *cen2-GFP* is a gift from A. Yamamoto and Y. Hiraoka. Other strains are our stock.

Biology Open • Supplementary information

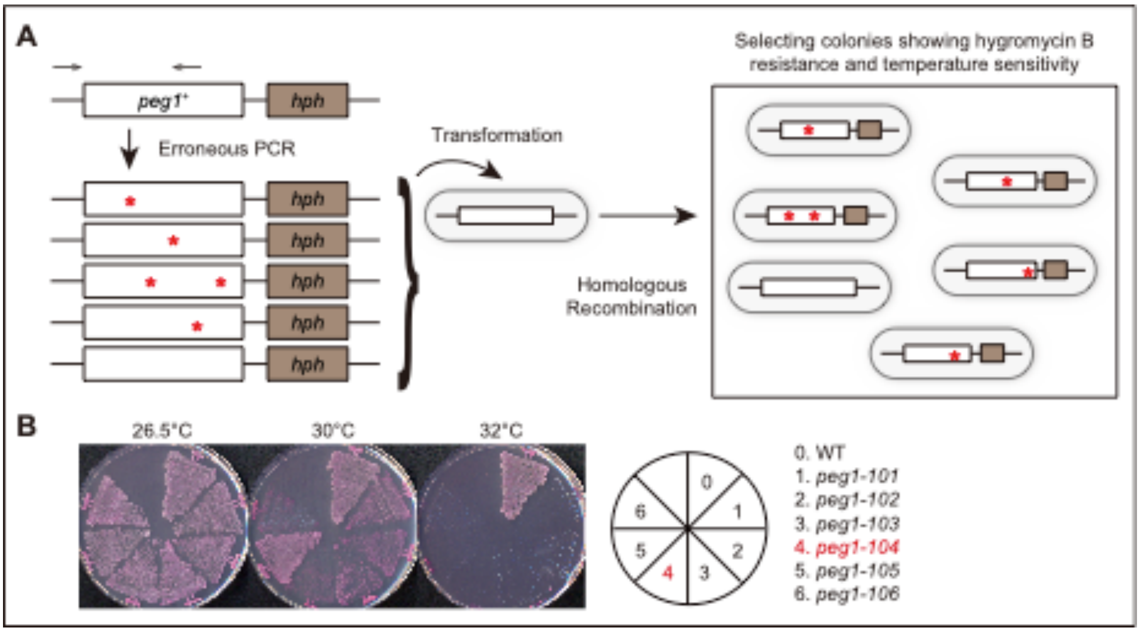

Figure S1: Isolation of *peg1* temperature sensitive mutants

(A) Schematics illustrating creation of temperature sensitive mutants of *peg1*. A DNA fragment containing the coding region of *peg1*$^{+}$ gene was amplified through erroneous PCR to induce random mutagenesis. Asterisks depict examples of point mutations introduced through PCR-based mutagenesis. Amplified fragments were introduced into WT cells to induce homologous recombination to replace the endogenous *peg1*$^{+}$ gene with the amplified products. Colonies showing hygromycin resistance and temperature sensitive growth defects were chosen as candidates.

(B) Reconfirmation of temperature sensitive growth defects of various *peg1* mutants isolated in the genetic screen in (A). *peg1-104* showed more severe growth defects than other candidates.

Biology Open • Supplementary information

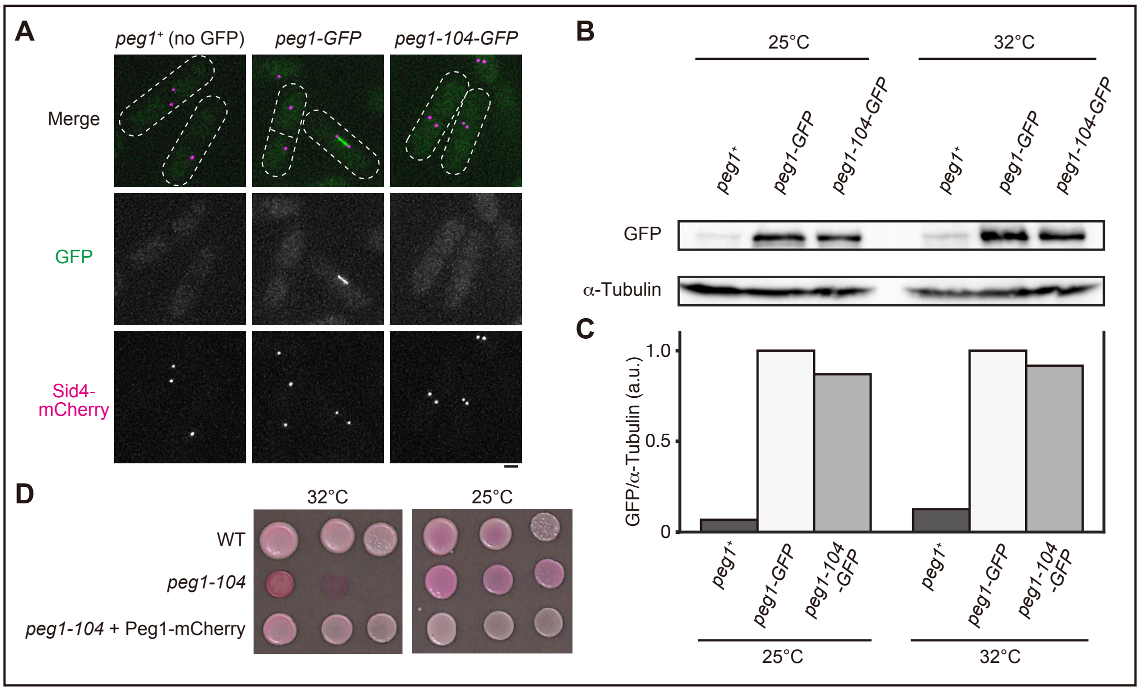

Figure S2: The mutant protein Peg1-104 does not localise to the spindle at the restrictive temperature

(A) Localisation of Peg1-GFP and Peg1-104-GFP shown together with the SPB marker Sid4-mCherry. Untagged *peg1+* cells are shown as a control. Dotted lines show the outline of individual cells. Cells with two SPBs (indicating the bipolar spindle) are in mitosis. Peg1-GFP localises to the spindle, whereas Peg1-104-GFP did not. In the strain expressing Peg1-GFP, the *GFP* gene was inserted at the 3'-end of the *peg1* coding region on the chromosome, therefore, the *peg1* gene was replaced with the fusion gene *peg1-GFP* at the original locus, so that Peg1-GFP could be expressed under the native promoter. In the *peg1-104-GFP* strain, the *GFP* gene was inserted at the 3'-end of the *peg1-104* coding region similarly. Scale bar, 2 µm. **(B, C)** An immunoblot analysis using the strains in (A). Cells were grown at the indicated temperatures. Proteins were extracted and subjected to SDS-PAGE, followed by immunoblotting using anti-GFP and anti-α-tubulin antibodies (B). Quantified intensities of bands corresponding to Peg1-GFP and Peg1-104-GFP normalised with those of α-tubulin (GFP/α-tubulin ratio) are compared in the graph (C). **(D)** Temperature-sensitive growth defects of *peg1-104* cells at 32˚C was suppressed by the fusion gene *peg1-mCherry*, which was ectopically expressed from an off-site chromosomal region (the *co2* locus; see **materials and methods**). Ten-fold serial dilution of cells with indicated genotypes were spotted on agar medium and incubated at 25˚C and 32˚C.

Biology Open • Supplementary information

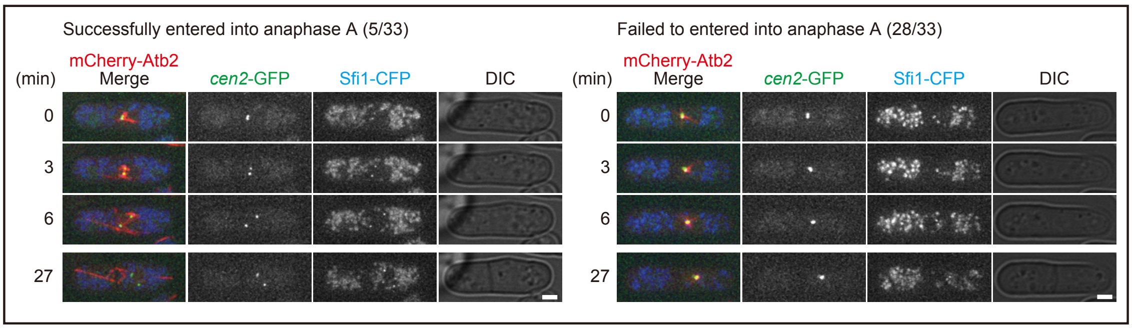

Figure S3: *peg1-104* cells often failed to undergo anaphase A

Related to **Figure 3A**, chromosome segregation defects of the *peg1-104* mutant were investigated in detail. mCherry-Atb2 (red), Sfi1-CFP (blue) and *cen2*-GFP (green) were observed at 32˚C. 33 of *peg1-104* cells were filmed from before SPB separation (0 min) to cytokinesis (indicated by the septum) every 3 min. Left: an example of cells segregated sister chromatids in anaphase A (5 cells), although all of them failed to deliver chromosomes into two daughter cells later in anaphase B. The defects in anaphase B could be due to failure in anaphase spindle organisation, as previously reported by Bratman and Chang (2007). Right: an example of the other 28 cells that failed to segregate sister chromatids.

Biology Open • Supplementary information

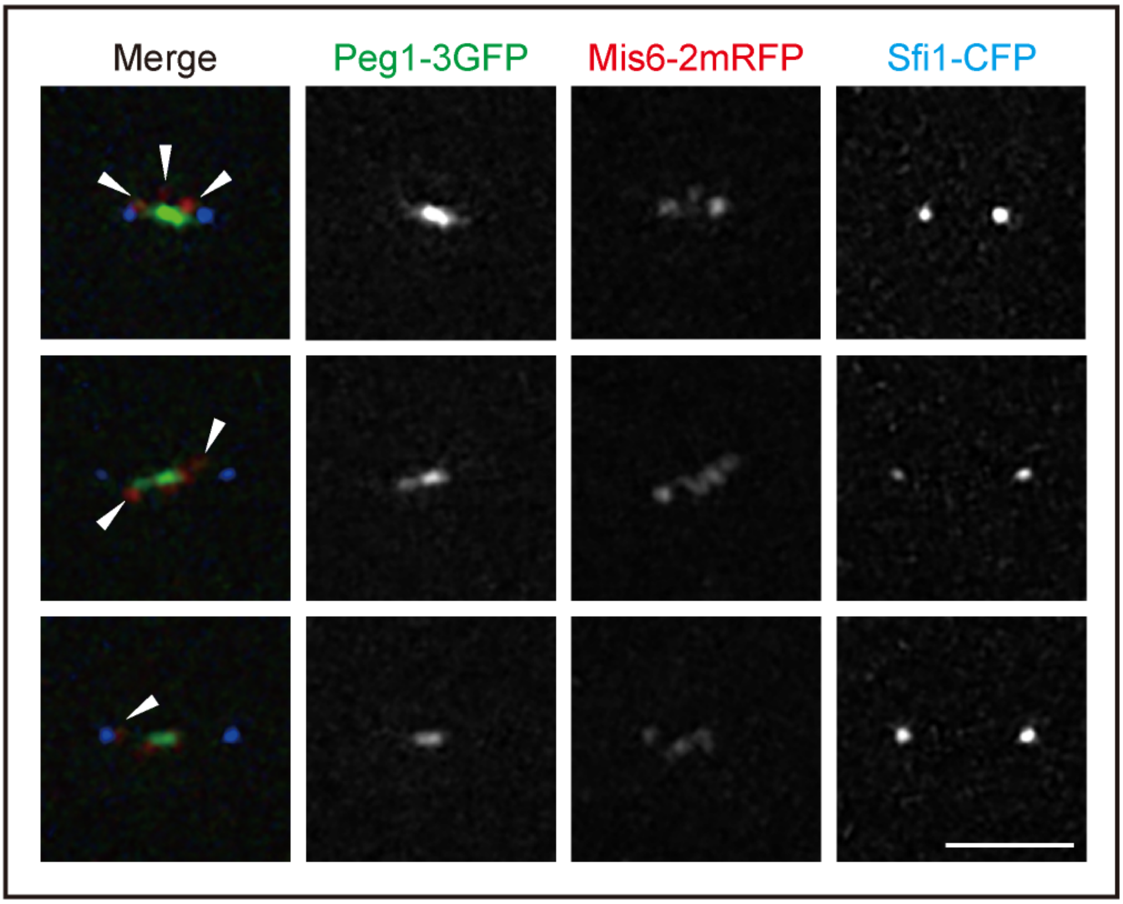

Figure S4: Peg1 does not localize to kinetochores

Fixed cells expressing Peg1-3GFP (green), Mis6-2mRFP (magenta) and Sfi1-CFP (blue) are shown. Arrowheads indicate Mis6-2mRFP dots which did not accompany Peg1-3GFP signal. Scale bar, 2 µm.

Biology Open • Supplementary information
